# Multiparametric dual-energy Computed Tomography in the diagnosis of lung adenocarcinoma patients and its correlation with tissue Ki-67 expression level

**DOI:** 10.4314/ahs.v25i2.38

**Published:** 2025-06

**Authors:** Gang Su, Hongwei Zhao

**Affiliations:** Department of Radiology, the Second Affiliated Hospital of Jiaxing University, Jiaxing, China

**Keywords:** Dual-energy CT, non-small cell lung cancer, adenocarcinoma, Ki-67 antigen

## Abstract

**Background:**

The purpose of this study was to investigate the variations in quantitative analysis of lung adenocarcinoma based on gender using dual-energy Computed Tomography (DECT), as well as its correlation with Ki-67, a proliferative marker.

**Methodology:**

A retrospective analysis was conducted on 79 patients diagnosed with lung adenocarcinoma, categorized into two groups based on gender. These patients underwent dual-energy CT scans, and the corresponding Ki-67 index was obtained. The study involved statistical analysis of general clinical characteristics, conventional chest imaging features, and dual-energy quantitative iodine values of the lung cancer patients in each gender group. Additionally, correlation analysis was performed to assess the relationship between the statistically significant parameters and Ki-67.

**Results:**

A significant disparity was observed in the values of virtual non-contrast (VNC) and Ki-67 between male and female patients with lung adenocarcinoma. Notably, male patients exhibited lower iodine concentration and a smaller increase in iodine value. Several quantitative iodine parameters showed a correlation with Ki-67. Among these parameters, OVERLAY_VP_ exhibited the strongest correlation. Distinct quantitative iodine parameters were identified for male and female lung adenocarcinoma patients, which exhibited a correlation with Ki-67, thereby facilitating the evaluation of the proliferative activity of lung adenocarcinoma.

**Conclusion:**

Dual-energy quantitative iodine parameters in lung adenocarcinoma patients exhibited gender-specific variations.

## Introduction

Lung cancer is the most prevalent malignant tumor in China, leading to high morbidity and mortality^1^. Among the various pathological subtypes, lung adenocarcinoma (ADC) is the most common^2^. The degree of malignancy and prognosis in lung cancer are associated with the tumor's pathological type and its proliferation index, with a higher proliferation index indicating a worse prognosis. Consequently, pre-operative imaging plays a crucial role in selecting the optimal therapeutic approach.

The nuclear protein Ki-67 serves as a vital indicator of the proliferation status of lung cancer cells, and its proliferation index (PI) is widely used as a marker for cell proliferation. While the Ki-67 index effectively reflects the metastasis and prognosis of lung cancer, assessing the proliferative activity typically requires invasive surgery or biopsy. Dual-energy computed tomography (DECT), among various imaging examinations, can differentiate between different tissue characteristics and provide both morphological and functional information.

Therefore, it offers a broader range of parameters compared to conventional chest-enhanced CT. Assessing the proliferative activity of tumor cells can be accomplished using Ki-67. However, limited research has been conducted on the correlation between Ki-67 and the quantitative iodine parameter of dual-energy CT in lung adenocarcinoma. This study aims to compare the imaging performance of lung adenocarcinoma patients of different genders, explore the diagnostic value of dual-energy CT in lung adenocarcinoma, and analyze the correlation between quantitative parameters and Ki-67 expression in lesion tissues. The findings will provide a theoretical foundation for the clinical diagnosis of lung adenocarcinoma.

## Materials and methods

### Patients

We conducted a retrospective analysis of patients diagnosed with lung adenocarcinoma who underwent dual-energy CT double-phase enhancement scans at our hospital between July 2019 and September 2020. The inclusion criteria for this study were as follows: (1) Patients had not received any prior treatment for lung cancer before the examination, (2) Patients had no known allergic reaction to contrast agents and were able to cooperate during the examination, and (3) Patients were confirmed to have lung adenocarcinoma through pathological examination. In the selected group of patients, there were 41 males, ranging in age from 42 to 80 years, with a mean age of 62 ± 1 years. The diameter of the lung lesions in males ranged from 12 to 61 mm, with a mean diameter of 25.44 ± 1.82 mm. Additionally, there were 38 females, ranging in age from 38 to 83 years, with a mean age of 65 ± 2 years. The diameter of the lung lesions in females ranged from 11 to 67 mm, with a mean diameter of 23.79 ± 1.81 mm. The study was approved by the ethics committee of Jiaxing Second Hospital Ethics Committee with the approval number JXEY-2019JX010.

### CT sign analysis

All the uploaded images were thoroughly examined by a highly experienced and board-certified chest radiologist with 15 years of clinical diagnostic expertise. The radiologist conducted a comprehensive assessment of the images without any knowledge of the patients' clinical or pathological data. During the evaluation, several factors were taken into consideration. The radiologist measured the diameter of the lesions and observed their distribution within the lungs, distinguishing between central and peripheral locations. Additionally, they looked for specific signs such as the lobar sign, which indicates the involvement of a particular lobe, and the burr sign, which refers to an irregular or spiculated lesion margin. The radiologist also checked for the pleural pull sign, adjacent bronchial truncation sign, and mediastinal or hilar lymph node enlargement sign. Furthermore, they assessed the enhancement characteristics of the lesions, paying particular attention to homogeneous tumor enhancement, defined as the absence of color variation in more than 90% of the observed areas on the venous phase pseudo-color images.

### DECT examination

All patients underwent preoperative scanning using a Siemens Healthcare SOMATOM Definition Flash CT scanner (Forchheim, Germany). The scans included both conventional chest scans and dual-energy enhancement scans in two phases: arterial and venous. The scan coverage extended from the thoracic inlet to the level of the suprarenal pole. Iohexol (350 mg/ml) was used as the contrast agent, administered intravenously at a dose of 60 to 100 mL (1.2 mL/kg body weight) with a flow rate of 3.5 ml/s, followed by a 30 mL saline flush at the same rate. The arterial phase scan commenced 5 seconds after contrast injection, while the venous phase scan was performed 100 seconds after injection, based on the distal attenuation of the thoracic aorta. The scan parameters were set as follows: matrix size of 512×512, A-bulb at 140 kV with 60 mAs, B-bulb at 80 kV with 214 mAs, and CAREDose 4D enabled. The collimator width was set at 60×0.6 mm, pitch at 0.7, and rotation time at 0.28 s. For reconstruction, the parameters used were a layer thickness of 3.0 mm, overlap of 0.7 mm, and reconstruction function D30f. Furthermore, iterative reconstruction technique was employed to reconstruct the data with a layer thickness of 1.0 mm.

### DECT image processing and quantitative analysis

The dual-energy data from the two phases were processed using the Siemens syngo.via workstation (version VB10B) and the dedicated Dual-Energy software with the Lung Nodules module. This software automatically subtracts the energy of bone and soft tissue to generate the iodine distribution map of the lesion. Manual region of interest (ROI) selection was performed by drawing the ROI on the most significant level and in close proximity to the central position of the lesion. Care was taken to avoid including cavities, necrotic tissue, calcifications, and blood vessels that could potentially affect the measurement results. All data were obtained using the mediastinal window settings, and efforts were made to ensure that the measurement position was consistent between the two phases. In cases where the lesion size was small, adjustments were made to the window width and position.

Quantitative iodine parameters, including the VN-C_AP_ in the arterial phase (VNCAP), the increased iodine value in the arterial phase (OVERLAY_AP_), the iodine concentration in the arterial phase (IC_AP_), the VNC in the venous phase (VNC_VP_), the increased iodine value in the venous phase (OVERLAY_VP_), and the iodine concentration in the venous phase (IC_VP_), were obtained. Each measurement was performed three times, and the average value was calculated.

### Pathological analysis

Ki-67 expression levels were assessed by pathologists with 15 years of experience in oncology using typical tumor samples collected from patients. Hematoxylin and eosin (HE) staining and immunohistochemical (IHC) staining were performed as routine procedures and the results were recorded. Ki-67 levels were detected using the internationally accepted two-step IHC method, and positive staining was indicated by brown-colored nuclei. The Ki-67 index was calculated as the percentage of positive cells observed under low to high magnification microscopy.

### Statistical analysis

All data were analyzed using Statistical Product and Service Solutions (SPSS) 22.0 software (IBM, Armonk, NY, USA). The χ2 test was employed to analyze differences in lesion location, morphological characteristics, and lesion enhancement characteristics in general data. Continuous data, such as patient age, lesion diameter, and two-phase CT quantitative iodine parameters of lesions, were assessed for normality. Normally distributed data were presented as Mean ± standard deviation (x̅±s), and differences between groups were analyzed using the two independent samples t-test. Non-normally distributed data were presented as median ± interquartile range (M ± Q), and differences between groups were analyzed using the rank sum test. Additionally, correlation analysis was performed to evaluate the relationship between the above parameters and Ki-67 expression, considering statistically significant differences. A significance level of P < 0.05 was considered statistically significant.

## Results

### Clinical Characteristics

Among the 79 cases of lung adenocarcinoma, 41 (41/79) were male and 38 (38/79) were female. The age of onset was slightly higher in females compared to males, but the difference was not statistically significant (Table 1).

**Table 1:** General clinical and imaging data of lung adenocarcinoma

Clinical characteristics		Male	Female	Statistical values	P-value
		(n=41)	(n=38)		
Age		61.80±1.38	65.05±1.65	t=-1.465	0.147
Lesion diameter		25.67±1.90	23.79±1.81	Z=-0.751	0.452
Lesion location (%)					
Central type		4 (3.9)	4 (5.2)	Chi-square=0.001	0.971
Peripheral type		37 (46.8)	34 (44.1)		
Morphological characteristics (%)					
With or without lobes	no	3 (3.8)	5 (6.3)	Chi-square=7.39	0.390
	yes	38 (48.1)	33 (41.8)		
With or without burr	no	13 (16.5)	11 (13.9)	Chi-square=0.071	0.790
	yes	28 (35.4)	27 (34.2)		
With or without pleural traction	no	13 (16.5)	10 (12.7)	Chi-square=0.278	0.598
	yes	28 (35.4)	28 (35.4)		
With or without adjacent bronchial truncation	no	25 (31.6)	28 (35.4)	Chi-square=1.443	0.230
	yes	16 (20.3)	10 (12.7)		
With or without mediastinal and hilar lymph node enlargement	no	27 (34.2)	26 (32.9)	Chi-square=0.059	0.080
	yes	14 (17.7)	12 (15.2)		
Reinforcement characteristics (%)					
Uniform reinforcement		8 (10.1)	10 (12.7)	Chi-square=0.519	0.471
Uneven reinforcement		33 (41.8)	28 (35.4)		

### CT Sign Analysis

The CT signs of the lesions in both groups are presented in Table 1. The lesion diameter tended to be slightly larger in males compared to females, but the difference was not statistically significant. Mediastinal and hilar lymph node enlargement was observed in 26 cases (32.9%). In terms of enhancement characteristics, 61 cases (77.2%) showed inhomogeneous enhancement, while only 18 (22.8%) showed homogeneous enhancement. However, there was no significant difference in the distribution of these CT signs between genders.

### Quantitative Iodine Parameters and Ki-67 Analysis of Adenocarcinoma

Table 2 compares the quantitative iodine parameters and Ki-67 indices of the lesions in the two groups. VNC_AP_ and VNC_VP_ were slightly higher in males compared to females, but the differences were not statistically significant. However, OVERLAY_AB_, IC_AB_ OVERLAY_VB_, and IC_VP_ showed statistically significant differences between the two groups. In both arterial and venous stages, females exhibited higher values of increased iodine value and iodine concentration compared to males. Additionally, Ki-67 expression was lower in females compared to males, and this difference was statistically significant.

**Table 2:** Comparison of quantitative iodine parameters and Ki-67 of plasma CT in male and female lung adenocarcinoma

Sex	Number of cases	VNC_AP_	OVERLAY_AP_	IC_AP_	VNC_VP_	OVERLAY_VP_	IC_VP_	Ki-67
Male	41	-5.10±44.79	24.58±16.55	0.90±0.74	-2.02±40.36	31.76±16.54	1.40±0.87	33±22
Female	38	-28.27±64.03	35.77±17.71	1.40±0.92	-26.15±65.42	47.39±16.68	1.96±0.89	15±10
Statistical value		t=1.851	t=-2.902	t=-2.660	t=1.955	t=-4.180	t=-2.829	Z=1.639
P-value		0.069	0.005	0.010	0.055	0.000	0.006	0.009

Note: VNC_AP_: virtual panning value in the arterial phase; OVERLAY_AP_: increased iodine value in the arterial phase; IC_AP_: iodine concentration in the arterial phase; VNC_Vp_: virtual panning value in the venous phase; OVERLAY_VP_: increased iodine value in the venous phase; IC_VP_: iodine concentration in the venous phase. The above parameters are normally distributed and are expressed as x̅ ± s. Two independent samples t-test was used. Ki -67: cell proliferation index was non-normally distributed data and non-parametric test was used.

### Correlation Analysis of Quantitative Iodine Parameters with Ki-67

Ki-67 showed a negative correlation with OVERLAY_AB_, OVERLAY_VB_, and IC_VP_ (P<0.05), while no significant correlation was observed with ICAP. Among the correlation coefficients, the highest correlation was found between Ki-67 and OVERLAY_VP_, while the lowest correlation was observed between Ki-67 and ICVP (Table 3).

**Table 3:** CT quantitative iodine parameters and Ki-67 correlation analysis for lung adenocarcinoma

Correlation	OVERLAY_AP_	IC_AP_	OVERLAY_VP_	IC_VP_
r-value	-0.353	-0.207	-0.356	-0.296
P-value	0.003	0.091	0.002	0.013

Note: OVERLAY_AP_: iodine increases value in arterial phase; IC_AP_: iodine concentration in arterial phase; OVERLAY_VP_: iodine increases value in venous phase.

IC_VP_: iodine concentration in the venous phase; Ki-67: cell proliferation index, for non-normally distributed data, analyzed by Spearman correlation.

## Discussion

Routine chest CT-enhanced examinations are currently the primary diagnostic tool for preoperative lung cancer diagnosis. The diagnosis involves observing the imaging features of the lesion on the plain lung window and assessing the degree of lesion enhancement, as well as mediastinal and hilar lymph nodes during the enhancement examination. Typical imaging manifestations of lung cancer include lobar signs, burr signs, and pleural pull signs. In our study, lobar signs (71/79), burr signs (55/79), and pleural pull signs (56/79) were observed. However, mediastinal and hilar lymph node enlargement and adjacent bronchial truncation signs were less common (26/79), likely due to the size and location of the lesions. It is possible that smaller lesions in the early stages of lung cancer may not exhibit lymph node enlargement. Furthermore, the predominance of non-uniform enhancement (61/79) in our study differs from previous reports, possibly due to variations in the observation method of enhancement. However, further investigation with larger sample sizes is required to confirm these findings^3^,^4^.

Our study revealed significant differences in dual-energy iodine parameters between males and females with lung adenocarcinoma. Although the VNC values in the arterial and venous phases should theoretically be the same, slight differences were observed in our data, possibly due to the removal of substances with similar atomic numbers to iodine during the dual-energy post-treatment process, resulting in iodine drift. Additionally, contrast agent interference may contribute to the observed differences. While the VNC values were higher in males compared to females, the differences were not statistically significant.

We also found that the values of increased iodine and iodine concentration were significantly higher in females compared to males in both arterial and venous stages. The difference was more pronounced in the venous phase, which may be attributed to the tumor's pathophysiology. Previous studies investigating the blood supply characteristics of solid lung nodules using dynamic enhancement CT have shown that tissue density changes after 100 seconds of contrast injection best reflect tissue blood flow characteristics. Therefore, we chose to begin the venous phase scan 100 seconds after contrast injection.

The Ki-67 index, which reflects the proliferative capacity of tumor cells, was assessed in our study. It is a nuclear antigen expressed specifically by proliferating cells and has been found to be associated with lung cancer development, metastasis, and prognosis. Higher Ki-67 index expression indicates increased proliferative activity of lung cancer cells and is associated with poorer prognosis and lower survival rates. Our findings indicated that Ki-67 index was negatively correlated with quantitative iodine parameters after enhancement^5^,^6^. This correlation may be attributed to enhanced proliferation of tumor cells with high Ki-67 expression, resulting in increased cell density and alterations in glandular tissue structure and function, ultimately leading to reduced tissue blood supply. Consequently, the increased iodine value and iodine concentration after enhancement were reduced. Previous studies by Huang Yanan et al. have reported varying Ki-67 expression in different pathological subtypes of lung cancer^7^. However, our study found significant differences in Ki-67 expression between males and females with the same adenocarcinoma, with higher expression observed in males. This finding suggests that the prognosis may be poorer in males compared to females, possibly due to the protective effect of estrogen^8^. Notably, Ki-67 is not an independent factor affecting the prognosis of non-small cell lung cancer patients, as it is correlated with other prognostic factors such as pathological type, clinical stage, and histological grading of the tumor itself. These factors warrant further investigation in future research^9^.

There are several limitations to our study. First, it was a retrospective and single-center study, which may limit the generalizability of the findings. Second, lung adenocarcinoma encompasses various pathological subtypes, such as adherent type, acinar type, papillary type, solid type, and micropapillary type, which are known to be associated with tumor prognosis. However, our study did not analyze the correlation among these subtypes. Lastly, our study focused solely on correlation analysis and did not include survival analysis.

## Conclusion

Dual-energy quantitative iodine parameters hold potential as imaging indices for predicting Ki-67 index expression levels in lung cancer. These parameters can provide valuable imaging-based predictions of lung cancer invasion and prognosis. However, further research with larger sample sizes and survival analysis is necessary to validate these findings.
